# Emerging roles of ADP-dependent glucokinase in prostate cancer

**DOI:** 10.1186/s40779-024-00518-7

**Published:** 2024-02-27

**Authors:** Xu Zhang

**Affiliations:** Department of Urology, the Third Medical Centre, Chinese PLA General Hospital, Beijing, 100039 China

**Keywords:** Prostate cancer (PCa), ADP-dependent glucokinase (ADPGK), Aldolase C, AMP-activated protein kinase (AMPK), Glycolysis

Currently, the standard clinical practice involves the predominant use of androgen deprivation therapy to treat advanced prostate cancer (PCa), which often inevitably progresses into castration-resistant PCa [1]. Because of its unfavorable prognosis and limited treatment alternatives, studies have emphasized the pressing requirement for novel therapeutic targets to enhance the efficacy of advanced PCa treatment and prolong patient survival.

Glucose metabolism plays a key role in tumor progression. Notably, the findings support the crucial roles of glucose metabolism, particularly the Warburg effect, in tumor cells, which enables tumor cells to prefer glycolysis even under aerobic conditions [2, 3]. The recognition of Warburg effect is evolving with increasing research in this field. This study by Xu et al. [3] started with a comprehensive bioinformatic analysis of 5 genes involved in glucose oxidative phosphorylation and identified ADP-dependent glucokinase (ADPGK) as a crucial participant in PCa, with its upregulation significantly correlating with adverse prognosis. Among the 5 enzymes responsible for the transition of glucose to glucose-6-phosphate, hexokinase 2 has been deemed a crucial factor for PCa regulation [4], whereas Xu et al. [3] identified ADPGK as a noncanonical kinase that promotes PCa progression for the first time. Because hexokinases are critical for glucose metabolism in normal tissues, ADPGK provides an ideal therapeutic target for cancer treatment. Furthermore, Xu et al. [3] revealed the intricate mechanisms by which ADPGK regulates PCa metabolic adaptability by interacting with aldolase C and activating AMP-activated protein kinase (AMPK) signaling to influence PCa proliferation and migration. Importantly, the ADPGK antagonist 8-Bromo-AMP showed a significant proliferation inhibition of PCa, which paves an avenue for future drug development and clinical trials.

In addition to tumor proliferation, tumor metastasis is a major cause of death in patients with cancer [5]. Therefore, finding ways to prevent cancer metastasis is a critical task in this field. Using integrative analyses and in vitro studies, a study demonstrated that a driver mutation of ADPGK accelerated breast cancer migration and metastasis [6]. Xu et al. [3] found that ADPGK overexpression significantly promotes PCa cell migration in vitro, which can be restrained by an ADPGK antagonist. Moreover, they used PC-3 xenograft mouse models and observed that ADPGK upregulation promoted tumor liver metastasis, indicating that targeting ADPGK could kill “two birds with one stone” by inhibiting tumor growth and metastasis. Accordingly, the authors comprehensively employed bioinformatics, clinical data, multiomics data, biochemical analyses, and molecular and cell biology to explore the critical roles of ADPGK during PCa progression, indicating that ADPGK is a significant risk factor and promoter for PCa.

Only a few studies have examined ADPGK. For example, Richter et al. [7] suggested that ADPGK expression is not affected by hypoxia, and Imle et al. [8] validated the crucial role of ADPGK in cell metabolism using a zebrafish model. Therefore, the findings presented in the research conducted by Xu. et al. [3] provided unique perspectives on the involvement of ADPGK in the metabolic mechanism of PCa, which may facilitate the development of innovative therapeutic approaches for PCa by targeting ADPGK as a promising treatment option.

Furthermore, aside from its important metabolic function in tumor cells, ADPGK has been reported to play essential roles in the regulation of immune cell activity, particularly in T cells [9]. PCa is a type of immune-cold tumor; however, its underlying mechanism remains unknown. Therefore, it is imperative to further investigate the effect of ADPGK on the development of a “cold” tumor immune microenvironment, and the novel findings will greatly affect the comprehensive management of PCa.

Based on previous basic research and clinical trials, effective treatment of PCa cannot be achieved using a single approach or target; therefore, a novel therapeutic regimen should be urgently developed to improve the survival and prognosis of patients with PCa. To date, multiple clinical trials of combination therapy for various PCa stages have been launched, including trials of abiraterone plus olaparib and androgen deprivation therapy plus docetaxel, which have shown promising clinical outcomes. Thus, collaborative strategies using different targets for effective PCa treatment will be the focus of future research and clinical applications. Ni et al. [10] reported combination immunotherapy using ADPGK neoantigen and nanovaccine in colorectal cancer and concluded that biadjuvant neoantigen nanovaccine is promising for optimizing personalized therapeutic neoantigen vaccines for cancer immunotherapy, highlighting the essential role of ADPGK in PCa immunotherapy as a neoantigen. Inspired by this, PCa cells can be effectively and specifically killed by 8-Bromo-AMP, and tumor cells with high ADPGK levels can be attacked by tumor vaccine-activated effector T cells. Thus, ADPGK potentially plays a dual role in inhibiting PCa progression as a drug target and neoantigen.

Moreover, the complex of ADPGK and aldolase C promotes the phosphorylation of AMPK; thus, a noncanonical kinase function of ADPGK was revealed in this study by Xu et al. [3]. The relationship between AMPK phosphorylation and the Warburg effect should be explored in the future.

In summary, Xu et al. [3] employed multiomic analyses to clarify the molecular mechanism of ADPGK in PCa progression. These findings significantly contribute to the advancement of our understanding of the tumorigenesis and development of PCa and provide promising therapeutic targets for future effective PCa treatment, which may fulfill the urgent requirement of managing PCa in the clinic.

## Data Availability

Not applicable.

## Abbreviations

PCa: Prostate cancer
ADPGK: ADP-dependent glucokinase
AMPK: AMP-activated protein kinase
